# On the Most Proper and Beneficial Application of Electricity in Diseases: Abstracted from the Essay on That Subject

**Published:** 1799-03

**Authors:** 

**Affiliations:** Munich


### *On the moſt proper and beneficial Application of Electricity in Diſeaſes: abſtracted from the Eſſay on that Subject, by Profeſſor* Imhof, *of Munich.*

It cannot be denied, that *medical electricity* has been only of late years reduced to a ſyſtematic form, and that we are in this reſpect particularly indebted to the labours of Tissot*, Cavallo, and Bertholon. The laſt

* The celebrated Tissot was unqueſtionably the firſt who treated ſcientifically on the medical application of electricity, exhibiting also juſt principles on which to found this proceſs, in his claſſical letter, '*De variolis, apoplexia et hydrope.*'

last of these meritorious characters framed a peculiar theory, according to which he derived all diseases either from the want or abundance of the electric fluid in the human body. He invented several useful instruments, and his method of applying them introduced a happy medium between the violent shocks recommended by some, and the timid practice of electrifying followed by others. By the exertions of such men, we at length arrived at stable principles, established on the broad basis of experience, by which we were taught, that electricity increases or promotes the circulation of the blood, and produces this effect particularly by what is called the *negative bath*. Thus we learned, by satisfactory proofs, that the electric fluid operates as a stimulating remedy on the animal body.

Meanwhile the well-known experiments of Galvani, with the metallic stimulus, excited an uncommon degree of attention in the medical world; but the consequent theories were too hastily formed, and pursued with hypothetical fallacy. This species of stimulus is certainly insufficient to afford a sure criterion of actual death, and to ascertain clearly the state of asphyxia, for this additional and obvious reason, that the disposition or tendency of the muscular fibre, to be affected to such a degree as to be thrown into convulsions by the application of metals, is considerably diminished in certain diseases, for instance, the gout and rheumatism; it must, however, be admitted that the nerves are very sensible electrometers, and that the animating principle, which pervades them, is forcibly stimulated in the transition of the electric fluid from one metal to another.

Electricity is one of the most powerful stimulating remedies which can be applied to the animal œconomy. Its effects may be considered both as general and local. Too violent shocks of it at once extinguish the vital principle, which, however, may be again kindled or excited by less powerful shocks. Hence the following positions may be admitted, in general, as correct and established; that electricity promotes the free circulation of the fluids and particularly the blood; that it accelerates perspiration, and increases animal heat, and likewise promotes all the secretions and excretions of the body*.

In the application of this powerful remedy, the following hints may be of service, as they are the result of actual experience, and not of speculation: 1. Electricity is attended with pernicious effects in *active* or *sthenic* diseases: 2. it is hurtful when, together with relaxation and debility, an uncommonly high

* It may properly be remarked here, that the effect of promoting the secretions and excretions by means of electricity, will then only take place when these have been diminished by atonic causes.

high degree of excitability in the organs of ſenſation is felt, as well as in thoſe of voluntary motion; and 3. if a preternatural impulſe of the fluids, ariſing from local irritation, prevail in any particular part of the body. In this caſe, electricity has a direct tendency to generate congeſtions, or the local accumulation of humours. In atonic collections of matter it is frequently found of ſervice, when the great vital activity of the ſolids alone is capable of reſolving the ſtagnations; but it is certainly detrimental, if the mechanical power of reſiſtance in the ſolid parts muſt, at the ſame time, be raiſed; and if the accumulated matter muſt be previouſly diminiſhed, before it can be diſcuſſed. Hence the application of electricity has ſometimes been highly beneficial in promoting a regular return of the menſes; but it has alſo, in certain caſes, been attended with dangerous effects.—It is further of conſiderable advantage in paſſive or aſthenic diſeaſes, particularly in caſes accompanied with a diminiſhed ſuſceptibility of ſtimuli in the organs of ſenſation and motion; provided that ſuch diſorders, at the ſame time, be manifeſt from the periodical returns of uncommon muſcular action, or by occaſional exceſs of the ſenſitive faculty in any particular part. Laſtly, the mode of imparting the electric fluid deſerves more attention than has hitherto been beſtowed upon it; and we ought never to communicate violent ſhocks, where leſs powerful ones might anſwer the purpoſe. Upon the whole, it appears to be an eſtabliſhed maxim that, under the circumſtances and conditions above ſpecified, both the electric bath, and the gentle application of the electric fluid to any particular part of the body, are always ſafe; and that the extraction of ſparks under ſimilar circumſtances is generally attended with advantage. The more violent methods of electrifying, on the contrary, have been productive of miſchief rather than good; ſo that they ought to be applied to thoſe individuals only, whoſe excitability is languid, or whoſe capacity for receiving impreſſions by external ſtimuli, is conſiderably diminiſhed.

